# DESIGNING INTERVENTIONS FOR OLDER ADULTS WITH LOW- AND MIDDLE-INCOME COUNTRIES SETTINGS IN MIND

**DOI:** 10.1093/geroni/igac059.818

**Published:** 2022-12-20

**Authors:** Zachary Baker, Manka Nkimbeng, Pearl G Cuevas, Ana Quiñones, Karmeet Kang, Joseph Gaugler, Ladson Hinton, Tetyana Shippee

**Affiliations:** Arizona State University, Tempe, Arizona, United States; University of Minnesota, Minneapolis, Minnesota, United States; Centro Escolar University, Manilla, Sta Rosa City, Laguna, Philippines; Oregon Health and Science University, Portland, Oregon, United States; Chitkara University, Rajpura, Punjab, India; University of Minnesota, Minneapolis, Minnesota, United States; University of California Davis, Sacramento, California, United States; University of Minnesota, Minneapolis, Minnesota, United States

## Abstract

Most people living with dementia (PLWD) live in low- and middle-income countries (LMIC) and the proportion in LMICs is poised to continue growing. But 99.9% of dementia funding is awarded to researchers in high-income countries (HIC). Our team of scientists from India, Cameroon, the Philippines, the USA, Ukraine, and Germany draw on our involvement in interventions in 12 countries to suggest one way to help meet the needs of PLWD living in LMICs. We suggest that researchers in HICs who are developing new interventions might consider the needs of LMICs during intervention development. By thinking through implementation scenarios in different settings or countries where barriers and facilitators to implementation vary in type, or in importance, it might speed future adaptation of those interventions to LMICs. We outline anticipated challenges, case studies from our own work, benefits for individual researchers, benefits for public health, and recommendations for employing this strategy.

